# Effects of Orange Juice Formulation on Prebiotic Functionality Using an *In Vitro* Colonic Model System

**DOI:** 10.1371/journal.pone.0121955

**Published:** 2015-03-25

**Authors:** Adele Costabile, Gemma E. Walton, George Tzortzis, Jelena Vulevic, Dimitris Charalampopoulos, Glenn R. Gibson

**Affiliations:** 1 Department of Food and Nutritional Sciences, The University of Reading, Reading, United Kingdom; 2 Clasado Research Services Ltd, Science and Technology Centre, Whiteknights, Reading, United Kingdom; National Institute of Agronomic Research, FRANCE

## Abstract

A three-stage continuous fermentative colonic model system was used to monitor *in vitro* the effect of different orange juice formulations on prebiotic activity. Three different juices with and without Bimuno, a GOS mixture containing galactooligosaccharides (B-GOS) were assessed in terms of their ability to induce a bifidogenic microbiota. The recipe development was based on incorporating 2.75g B-GOS into a 250 ml serving of juice (65°Brix of concentrate juice). Alongside the production of B-GOS juice, a control juice – orange juice without any additional Bimuno and a positive control juice, containing all the components of Bimuno (glucose, galactose and lactose) in the same relative proportions with the exception of B-GOS were developed. Ion Exchange Chromotography analysis was used to test the maintenance of bimuno components after the production process. Data showed that sterilisation had no significant effect on concentration of B-GOS and simple sugars. The three juice formulations were digested under conditions resembling the gastric and small intestinal environments. Main bacterial groups of the faecal microbiota were evaluated throughout the colonic model study using 16S rRNA-based fluorescence in situ hybridization (FISH). Potential effects of supplementation of the juices on microbial metabolism were studied measuring short chain fatty acids (SCFAs) using gas chromatography. Furthermore, B-GOS juices showed positive modulations of the microbiota composition and metabolic activity. In particular, numbers of faecal bifidobacteria and lactobacilli were significantly higher when B-GOS juice was fermented compared to controls. Furthermore, fermentation of B-GOS juice resulted in an increase in *Roseburia* subcluster and concomitantly increased butyrate production, which is of potential benefit to the host. In conclusion, this study has shown B-GOS within orange juice can have a beneficial effect on the fecal microbiota.

## Introduction

The concept of modulating gut health through diet is not new and dates back to at least the beginning of the 20th century. More recently, sound scientific rationales have been proposed and investigated [1].

Dietary substrates reaching the large intestine are able to influence the composition and activities of indigenous bacteria through their fermentation capacities [2, 3]. Prebiotics, specifically inulin-type fructooligosaccharides (FOS) and galactooligosaccharides (GOS) are known to support the growth of beneficial bacteria, such as bifidobacteria, and numerous intervention studies have shown such properties *in vivo* [4, 5].

Since 2006 much research has implicated the gut microbiota and the regulation of host immunity in the development of different conditions such as metabolic syndrome and associated disorders [6,7]. Therefore, through studying the gut microbiota in metabolic disease and the subsequent impact of prebiotics potential future new therapeutic approaches may be developed. Galacto-oligosaccharides are prebiotics that have been shown in human feeding studies to selectively stimulate the growth of bifidobacteria [8–11] and lactobacilli. Moreover, human studies on infants have shown GOS to offer benefits in terms of stool consistency, while increasing bifidobacterial numbers [12,13] in adults, GOS have also been seen to alleviate symptoms of irritable bowel syndrome [8].

Recently, much attention has been focused on the properties of prebiotics to be incorporated into food products whilst not negatively impacting on organolectic properties and maintaining stablity during food processing. There is a considerable amount of information on the stability of prebiotics, in particular, GOS, FOS and inulin, mainly from experiments using model systems and to a lesser extent with real foods. GOS in general are very stable under acidic conditions and high temperatures and for this reason they can be potentially added to a variety of acid or heated foods, such as yogurts, fermented milks, buttermilk, pasteurised fruit juices and bakery products [14,15].

The stability of gal-polyols and oligosaccharides during pasteurization at a low pH in various fruit juices has also been demonstrated; in most cases, more than 99% of GOS survived pasteurization [16]. To date, the reason for the high stability of GOS is the presence of b-linkages, although other factors, such as the sugar residues present, the ring form and the anomeric configuration can play a role too [16].

Controversially, inulin and FOS have been suggested to be less stable than other oligosaccharides at conditions of low pH and high temperatures.

Suggested further work in the topic includes the evaluation of the stability of established prebiotics in real food systems rather than model solutions, as there is a lack of data on this and the scarce existing data suggest that the food matrix can influence prebiotic stability. A lack of data exists also in regard to emerging prebiotics. Another important technological aspect of prebiotics is their effect on the physicochemical and organoleptic properties of the food product.

In respect to GOS, there are few commercial examples of GOS-containing food products, and very little information on the impact of GOS on the physicochemical properties of the food carrier.

In the current study, three foods were developed, a novel prebiotic-fortified juice, a juice with matching sugar concentration and a placebo juice. Post-processing, the carbohydrate concentrations (simple sugars and added prebiotic) were assessed via ion-exchange chromatography. By using initial pre-digestion under similar conditions to the upper gastrointestinal tract partially digested foods likely to persist to the colon can be investigated. In the present study, the impact of the three juice products on the human intestinal microbial ecosystem was investigated using an *in vitro* three-stage continuous culture system which simulated the human large intestine (colon model). The colonic model provides a controlled environment to assess microbial changes and organic acid production. The main bacterial groups of the faecal microbiota were evaluated during the study using 16S rRNA-based fluorescence in situ hybridization (FISH) approach.

## Materials and Methods

### Galactooligosaccharide mixture (B-GOS)

A prebiotic GOS mixture (B-GOS; BiMuno) was provided in powder form and supplied by Clasado Ltd, Milton Keynes, United Kingdom. The product was in dry powder form consisting of (wt:wt) 48% GOS with a degree of polymerization between 2–5, 22% lactose, 18% glucose and 12% galactose.

### Preparation of orange juices

The orange juices were prepared in batches of 500ml, which is equivalent to two servings of juice. To produce the juices, an orange juice concentrate was sourced from Minute Maid, (Coca-Cola, London, UK) and diluted with either water, a BiMuno solution or a solution of mixed carbohydrates (glucose, galactose, maltodextrin, lactose), to achieve a Brix value of 11.2°C for each orange juice; the Brix value of the juice concentrate was 65°C. In Table 1 the nutritional composition in 240 ml is reported. The target concentration of BiMuno in the B-GOS juice was 5.5g per 250ml serving, i.e. 2.75g B-GOS. The composition of the three produced orange juices is reported in Table 2. The juices were batch pasteurized at 65°C for 30 minutes and then freeze dried.

**Table 1:** Nutritional composition in 240mL serving size.

Amount Per Serving Calories 110	
	% Daily Value^+^,*
Total Fat 0g	0%
Sodium 15mg	1%
Potassium 450mg	13%
Total Carb 27g	9%
Sugars 24g	
Protein 2g Not a significant source of protein	
Vitamin C 130%	Calcium 2%
Thiamin 10%	Niacin 2%
Vitamin B6 4%	Folate 15%
Magnesium 6%	

^+^ Not a significant source of calories from fat, saturated fat, trans fat, cholesterol, dietary fiber, vitamin A and iron;

* Percent Daily Values are based on a 2,000 calorie diet.

**Table 2:** Ingredients for orange juice preparation.

	Control	B-GOS Juice	Mixed sugar juice
Water (mL)	417	417	417
Concentrate (mL; 65°Brix)	83	83	83
BiMuno (g)	0	11	0
Glucose (g)	0	0	0.73
Galactose (g)	0	0	0.48
Maltodextrin (g)	0	0	1.21
Lactose (g)	0	0	8.6

### Analysis of B-GOS

To determine whether the sugar concentrations were maintained after the juice production processes, the AOAC 2001.02 (method 32–33) based on the methodology described by de Slegte [17]. The carbohydrate composition of the reaction mixture was determined by high performance anion exchange chromatography coupled with pulsed amperometric detector (HPAEC-PAD). A Dionex system (Dionex corporation, Surrey, UK) consisting of a GS50 gradient pump, an ED50 electrochemical detector with a gold working electrode, an LC25 chromatography oven, and an AS50 autosampler was used. Separation was performed using a pellicular anion-exchange resin based column, CarboPac PA-1 analytical (4mm×250 mm), connected to a CarboPac PA1 Guard (4mm×50mm) (Dionex corporation, Surrey, UK). The column was maintained at 25^°^C; elution was performed at a flow rate of 1ml/min using gradient concentrations of sodium hydroxide and sodium acetate solutions. All chromatographic analyses were performed in triplicate.

### Simulated human digestion of juice (from mouth to small intestine)

Frozen juice samples of orange juices were thawed and 60g of each sample were digested by an *in vitro* simulation of the upper gut digestion. The remaining solution was dialysed with a membrane of 100–200 Daltons cut off (Spectra/por 100–200 Da MWCO dialysis membrane, Spectrum Laboratories Inc., UK) to remove monosaccharides from the digested juices and the retentate, the product was then freeze dried for use in the *in vitro* systems as described by Maccaferri *et al*. [18].

### Collection and stool sample preparation

Faecal samples were obtained from volunteers at risk of metabolic syndrome (two males, one female; age 30 to 38 years; BMI: 18.5–25). Faecal samples were collected on site, kept in an anaerobic cabinet (10% H_2_, 10% CO_2_ and 80% N_2_) and used within a maximum of 15 minutes following voiding. Samples were diluted 1/10 w/w in anaerobic PBS (0.1 mol/L phosphate buffer solution, pH 7.4) and homogenized (Stomacher 400, Seward, West Sussex, UK) for 2 minutes at 240 paddle beats per minute.

### Three-stage continuous culture colonic model system

The three-stage continuous culture model of the human colon comprised of 3 glass fermenters of increasing working volume, simulating the proximal (V1, 280 mL), transverse (V2, 300 mL) and distal colon (V3, 320 mL). The 3 fermenters, connected in series, were kept at 37°C, pH was maintained at 5.5 (V1), 6.2 (V2) and 6.8 (V3) and anaerobic conditions were introduced by continuously sparging with O_2_-free N_2_ at a rate of 15 mL/min. The fermentation system was designed and validated to reproduce the spatial, temporal, nutritional, and physicochemical characteristics of the microbiota in the human colon [19]. V1 was fed by means of a peristaltic pump with a culture medium previously described [18] and consisting of the following chemicals (g/L) in distilled water: starch, 5.0; pectin (citrus), 2.0; guar gum, 1.0; mucin (porcine gastric type III), 4.0; xylan (oatspelt), 2.0; arabinogalactan (larch wood), 2.0; inulin, 1.0; casein (BDH Ltd.), 3.0; peptone water, 5.0; tryptone, 5.0; bile salts No. 3, 0.4; yeast extract, 4.5; FeSO_4_. 7H2O, 0.005; NaCl, 4.5; KCl, 4.5; KH_2_PO_4_, 0.5; MgSO_4_.7H_2_O, 1.25; CaCl_2_.6H_2_O, 0.15; NaHCO_3_, 1.5; cysteine, 0.8; hemin, 0.05; Tween 80, 1.0.

Each stage of the colonic model was inoculated with 100 mL faecal slurry. The total system transit time was 48 h. Following inoculation, the colonic model was run as a batch culture for 24 h in order to stabilise the bacterial populations prior to the initiation of medium flow. After 24 h (T0), the medium flow was initiated and the system ran for 8 full volume turnovers to allow for steady state to be achieved (SS1) (assessed through stabilisation of the SCFA profiles (+/-5%). Taking into account, the operating volume (900 mL) and the retention time (48 h) of the colonic model system, the dialysis retentate of the different juices was added daily into V1 at 1% (w/v). The tested juices were added to the system for a further 8 volume turnovers upon which steady state 2 (SS2) was achieved.

### *In vitro* enumeration of bacteria population by FISH

Numbers of the 9 main intestinal bacterial groups, as well as total bacterial populations, were evaluated by FISH analysis, as previously described by Martin-Pelaez and colleagues [20]. The probes used are reported in Table 3 [22–27] and were commercially synthesized and 5’-labelled with the fluorescent Cy3 dye (Sigma-Genosys, UK).

**Table 3:** Oligonucleotide probes used in this study for FISH analysis.

Probe	Target group	Reference
EUB338^a^	Most bacteria	[22]
EUB338II^a^	Most bacteria	[22]
EUB338III^a^	Most bacteria	[22]
Bac303	*Bacteroides* spp.	[23]
Bif164	*Bifidobacterium* spp.	[23]
Lab158	*Lactobacillus-Enterococcus* spp.	[24]
Erec482	Most of the *Clostridium coccoides*-*Eubacterium rectale* group (*Clostridium* cluster XIVa and XIVb)	[25]
Chis150	*Clostridium histolyticum* group	[26]
Prop853	*Clostridium* cluster IX	[27]
Rrec 584*2*	*Roseburia* subcluster	[28]
Fprau655	*Faecalibacterium prausnitzii and related sequences*	[28]

^a^, ^b^These probes are used together in equimolar concentrations

### Short chain fatty acids (SCFAs) analysis by gas chromatography

Aliquots of 1 ml collected from each vessel in microcentrifuge tubes were centrifuged at 13000 g for 5 min. The supernatants were transferred into fresh microcentrifuge tubes and stored at -20°C until use. Samples were derivatized based on the method of Richardson *et al*., [21]. The supernatants stored at -20°C were thawed on ice and centrifuged at 13000 g for 10 min. 500 μL of each supernatant were transferred into fresh microcentrifuge tubes and 25 μL of internal standard (2-ethyl butyric acid) followed by 250 μL of concentrated HCl and 1 mL of ether added to each tube. Tubes were vortexed for 1 min and centrifuged at 3000 g for 10 min. The top ether layer was collected and transferred into fresh microcentrifuge tubes. Aliquots (400μL) of the ether extract were pipetted into a Wheaton vial and then 50 μL of N-tertButyldimethyl silyl N-methyltrifluoroacetamide (MTBSTFA) was added. The vials were sealed tightly by screwing after addition of MTBSTFA and heated at 80°C for 20 min in a water bath. Samples were transferred to Agilent crimp cap vials for gas chromatography analysis. Vials were capped with Crimp top natural rubber/PTFE seal type 7 aluminium silver 11 mm Chromacol caps and sealed using a crimper. The capped vials were left at room temperature for 48 h for derivatization. Calibration was achieved using standard solutions of derivitized acetic, propionic, i-butyric, n-butyric, i-valeric, n-valeric, and n-caproic acids as described for the test samples. The final concentration of each standard was 25, 10, 5, 1, and 0.5 mM. The derivatized samples were run through a 5890 series II GC system (HP, Crawley, West Sussex, UK) fitted with SGE-HT5 column (0.32 mm × 25 m × 0.1 μm; J&W Scientific, Folsom, CA, USA) and flame ionisation detector. Helium was used as the carrier gas and was delivered at a flow rate of 14 mL/min. The head pressure was set at 10 psi with a split ratio of 10:1. Injector, column and detector were set at 275, 250 and 275°C respectively. One micro liter quantity of each sample was injected with a run time of 10 min. Peaks were integrated using the Atlas Lab managing software (Thermo Lab Systems, Mainz, Germany). Organic acid concentrations were quantified by calculating the relative response factors as compared to the internal standards and calculating concentrations within the samples accordingly.

### Statistical analysis

Data were analyzed by one-way ANOVA, using Tukey’s post-test analysis when the overall P value of the experiment was below the value of significance (P < 0.05). An additional paired t-test was applied in order to assess the significance of results of single pairs of data. Analyses were performed using GraphPad Prism 5.0 (GraphPad Software, La Jolla, CA, USA).

## Results

### Analysis of B-GOS in juices

On average, the quantity of B-GOS recovered in B-GOS-juice was approximately 0.022g per ml, indicating that that 250ml provides 2.75g of B-GOS. Results therefore show that no loss of B-GOS was encounterd during the food processing or storage.

### Effects of different juices on prebiotic functionality in colonic model

**Fig 1:**
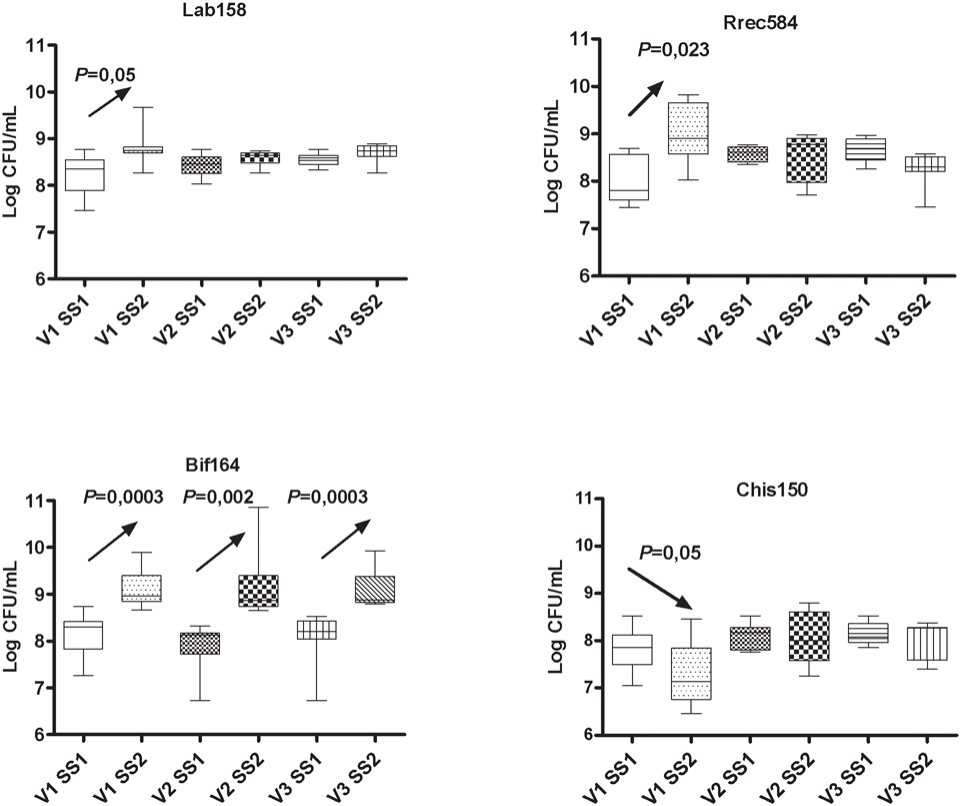
Bacterial groups detected by FISH in culture broths recovered from vessels (V1, V2 and V3) of an *in vitro* colonic model before (SS1) and after (SS2) daily administration of B-GOS juice. Results are reported as mean of the data of three colonic models (Log10 CFU/mL) ± standard deviations (SD).

**Fig 2:**
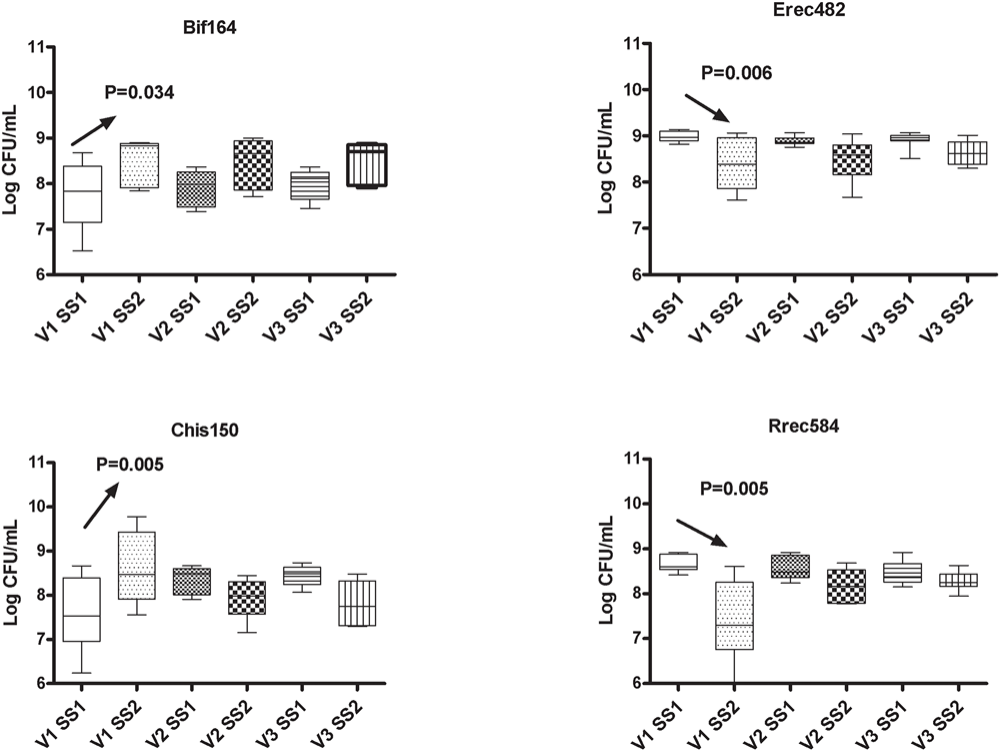
Bacterial groups detected by FISH in culture broths recovered from vessels (V1, V2 and V3) of an *in vitro* colonic model before (SS1) and after (SS2) daily administration of mixed sugar juice. Results are reported as mean of the data of three colonic models (Log10 CFU/mL) ± standard deviations (SD).

The human intestinal microbiota was assessed before and after supplementation with B-GOS juice, a control juice which had no added prebiotic, and a positive control juice which contained a mixed sugar concentrate to provide carbohydrates equivalent to the B-GOS juice. The administration of control juice did not mediate any significant change in the concentrations of the bacterial groups that were investigated (Supplementary material). On the other hand, the B-GOS juice mediated a significant increase in numbers of bacteria within the *Bifidobacterium* genus (detected by Bif164) in all the stages of the colonic model system (from 8.15 to 9.10 log CFU/ml in V1 [P = 0.0003], from 7.91 to 9.14 log CFU/ml in V2 [P = 0.003]) and from 8.10 to 9.09 CFU/ml [P = 0.0029] in V3) whereas the lactic acid bacteria group (LAB) significantly increased in V1 (from 8.24 to 8.81 log CFU/ml in V1 [P = 0.05]. Moreover, an overall decrease of the *Clostridium* cluster I and II (detected using Chis 150) was observed in vessel 1 (from 7.83 to 7.29 log CFU/ml in V1 [P = 0.042]), whereas the *Roseburia/E*. *rectale* groups increased from 8.04 to 9.02 log CFU/ml in V1 [P = 0.0023], from 8.05 to 8.49 log CFU/ml in V2 [P = 0.002]) (Fig. 1). Similar to B-GOS juice, the mixed sugar juice induced a significant increase in bifidobacteria in vessel 1 (from 7.77 to 8.53 log CFU/ml in V1 [P = 0.034]. Furthermore, an overall decrease of the *E*. *rectale/Clostridium* cluster XIVa group V1 was observed following supplementation of mixed sugar juice (from 8.98 to 8.34 log CFU/ml in V1 [P = 0.05], as well as a decrease in *Roseburia/E*. *rectale* groups (from 8.68 to 7.40 log CFU/ml in V1 [P = 0.048]) (Fig. 2).

### Short chain fatty acid analysis

Short chain fatty acids (SCFAs), which are the principal end products of gut bacterial metabolism, were measured in the three different vessels of the colonic model systems, at SS1 and SS2 by gas chromatography (Table 4). The administration of B-GOS juice in V1 induced a significant increase in butyrate (29.93 to 41.43 mM; P<0.001) and acetate (from 38.79 to 60.94 mM; P = 0.002) from SS1 to SS2 (Table 4). The administration of mixed sugar juice in V1 induced a significant decrease in butyrate (49.93 mM in SS2 to 40.14 mM in SS1, P = 0.002) (Table 4). When mixed sugar juice was added into the colonic system, the acetate levels increased significantly in V1, from SS1 to SS2 (Table 4, P = 0.002). None of the changes in the concentrations of SCFAs in all vessels at SS2 were significant with the control juice when compared to SS1 (Table 4), which is in accordance with the microbiological data.

**Table 4:** Short-chain fatty acids concentrations in culture broths recovered from vessels (V1, V2 and V3) of an *in vitro* colonic model before (SS1) and after (SS2) the daily administration of B-GOS containing juice, mixed sugar juice and control juice, as assessed by GC analysis.

Metabolites	Vessel 1 (V1)		Vessel 2 (V2)		Vessel 3 (V3)	
	Before B-GOS orange Juice(SS1)	After B-GOS orange Juice (SS2)	Before B-GOS orange Juice(SS1)	After B-GOS orange Juice (SS2)	Before B-GOS orange Juice(SS1)	After B-GOS orange Juice (SS2)
Acetate	38.79 ± 5.78	60.94 ± 1.79^a^	69.23 ± 3.13	79.67 ± 6.20	68.01 ± 2.14	70.70 ± 8.24
Propionate	57.45 ± 1.77	63.09 ± 2.33	55.51 ± 2.55	62.69 ± 5.76^a^	69.31 ± 4.89	70.71 ± 2.69
Butyrate	29.93 ± 11.53	40.43 ± 3.29^a^	52.04 ± 6.70	50.12 ± 6.85	62.97 ± 5.29	61.58 ± 2.05
**Metabolites**	**Vessel 1 (V1)**		**Vessel 2 (V2)**		**Vessel 3 (V3)**	
	**Before Mixed sugar orange Juice(SS1)**	**After mixed sugar orange Juice (SS2)**	**Before Mixed sugar orange Juice(SS1)**	**After mixed sugar orange Juice (SS2)**	**Before Mixed sugar orange Juice(SS1)**	**After mixed sugar orange Juice (SS2)**
Acetate	48.79 ± 5.78	60.92 ± 1.79^b^	59.10 ± 2.13	79.67 ± 6.20	62.08 ± 1.98	70.70 ± 8.24
Propionate	47.45 ± 1.77	53.05 ± 2.10	51.42 ± 1.87	56.69 ± 4.56^a^	59.28 ± 3.89	64.71 ± 1.69
Butyrate	49.93 ± 11.53	40.14 ± 2.34 ^b^	54.03 ± 3.70	48.91 ± 5.78	52.97 ± 4.28	59.88 ± 5.05
**Metabolites**	**Vessel 1 (V1)**		**Vessel 2 (V2)**		**Vessel 3 (V3)**	
	**Before control orange Juice(SS1)**	**After control orange Juice (SS2)**	**Before control orange Juice(SS1)**	**After control orange Juice (SS2)**	**Before control orange Juice(SS1)**	**After control orange Juice (SS2)**
Acetate	52.79 ± 3.68	59.94 ± 1.79	69.23 ± 3.13	72.67 ± 5.20	78.01 ± 2.14	80.70 ± 8.24
Propionate	44.38 ± 1.77	52.19 ± 1.33	55.51 ± 2.55	62.69 ± 5.76^a^	69.31 ± 4.89	74.71 ± 2.69
Butyrate	53.43 ± 8.43	47.43 ± 3.29	52.04 ± 6.70	55.12 ± 7.85	62.97 ± 5.29	61.58 ± 2.05

For each sample, measurements were performed in triplicate. Results are means (mM) of the measurements in the two colonic models ± standard deviation (SD).

^a^Designates significant differences between SS1 and SS2 at confidence level of *P*<0.05

^b^Designates significant differences between SS1 and SS2 at confidence level of *P*<0.05.

## Discussion

A prebiotic juice was developed in the current study and using *in vitro* assessment the prebiotic functionality of the final product was maintained, as compared to control products. The juice development was based on incorporating 2.75g B-GOS into an acceptable serving of orange juice. The Bimuno product naturally contains about 52% simple sugars, these would normally be digested in the upper gastrointestinal tract, therefore just the B-GOS would persist to the large intestine, to create an effective control for this *in vitro* work a product with match sugar concentration was developed, furthermore, a placebo juice, without these additional sugars was also developed. Therefore with optimal pre-digestion the control products would be expected to perform similarly in the model systems and lead to no changes between steady state 1 and 2.

B-GOS juice induced positive modulations of the microbiota composition and metabolic activity. In fact, B-GOS juice mediated significant increases of *Bifidobacterium* spp. in all three vessels of the model system. Bifidobacteria is a genera associated with many positive effects including immunomodulation and reducing IBS symptoms [23, 8, 9]. As such, this change is regarded as a potential benefit. This bifidogenic (prebiotic) effect has been observed on consuption of B-GOS, whilst not within a matrix [8– 10]. The mixed sugar juice also led to increases in bifidobacteria in the first vessel of the model system, but this change was of a smaller magnitude to that seen with the B-GOS juice, thus indicating the additional ability of B-GOS to positively modulate the microbiota within the juice matrix. The bifidogenic effect of the mixed sugar juice, and not the control juice, indicates that sugars used in juice manufacture were not completely removed in the pre-digestion process and are therefore available for fermentation; this is unlikely to be the case *in vivo*. The control juice did not lead to any significant changes to the microbiota, thus indicating a lower persistence of the sugars through the initial *in vitro* predigestion compared to the other juice preparations. Lactic acid bacterial numbers increased significantly in vessel 1 of the B-GOS juice supplemented model; this potentially beneficial effect was not observed following fermentation of the mixed sugar juice, thereby showing an enhanced effect of the B-GOS juice. Similarly in human studies B-GOS supplementation has led to increased levels of lactobacilli [9]. The bifidobacterial and lactobacilli changes show that the prebiotic functionality of the B-GOS was maintained post product processing.

Further microbial effects observed upon fermentation of the B-GOS juice, but not the mixed sugar juice, included a decrease in the *C*. *histolyticum* group. This group, although present in the healthy gastrointestinal tract, contains some known potential pathogens, therefore a decrease in numbers is seen to be of benefit. Indeed, Vulevic *et al*., [9] also observed B-GOS to impact on this microbial group. In addition to this, the mixed sugar juice fermentaion led to an increase in this microbial group, which is indicative of the non-selective fermentation nature of the sugars within this particular preparation.

B-GOS juice also lead to additional increases of the *Roseburia* subcluster, a change correlated with a potentially beneficial butyrogenic effect following B-GOS juice administration. Butyrate is associated with many benefits in terms of colonic health [4]. For example, it is the preferred energy source for colonic epithelial cells, and promotes normal cell differentiation and proliferation. Additionally, both the B-GOS juice and the mixed sugar juice fermentation generated increases in acetate. The metabolic fate of acetate is within the muscles, kidney, brain and heart [28] Furthermore, bifidobacteria are acetate producers, but do not form butyrate, therefore it is possible that butyrate was produced by acetate utilisation, by bacteria in the *Roseburia* group [29]. SCFAs, in addition, help to regulate sodium and water absorption, and can enhance absorption of calcium and other minerals as well as lower the colonic pH. This latter modification has the potential to inhibit growth of putative pathogens providing an additional potential benefit to the host [30, 31, 32]. Within this study the faecal samples were collected from individuals with metabolic syndrome, to see if this product could be appropriate for modulating the microbiota in those at risk of developing cardiovascular disease and diabetes. As this group of individuals has been considered to have a modified microbiota that may benefit from increased bifidobacteria levels [33]. The results of this *in vitro* work show positive modulations of the microbiota and indicate that more work with B-GOS juice in those at risk of metabolic syndrome would be appropriate to determine if microbial changes can improve other associate metabolic syndrome parameters, as seen in murine studies [34].

Overall the findings showed that functionaility of B-GOS was maintained within the B-GOS juice, therefore supporting the utilization of prebiotic enriched juice for potentially improving gastrointestinal health. Further work is however, required to assess if this functionality is also maintained *in vivo*, within a population at risk of metabolic syndrome.
